# Supplementary material for: Short-term outcomes after emergency surgery for complicated peptic ulcer disease from the UK National Emergency Laparotomy Audit: a cohort study
Source: BMJ Open. 2018 Aug 20;8(8):e023721. doi: 10.1136/bmjopen-2018-023721 (PMC6104767; doi:10.1136/bmjopen-2018-023721)
Supplement: Supplementary data [file bmjopen-2018-023721supp001.pdf]

SUPPLEMENTARY MATERIAL

Supplementary table 1. Grouping of categorical variables for analysis.

Variable	Group	Original values
ASA	1	1
	2	2
	3	3
	4 & 5	4
		5
Peritoneal contamination type	None / minimal	None / serous
		Gas / minimal
	Significant	Pus
		Bile
		Gastro-duodenal contents
		Small bowel contents
		Faeculent fluid
		Faeces
		Blood
Peritoneal contamination extent	None / single quadrant	None
		Single quadrant
	Multiple quadrants	Multiple quadrants
Preoperative CT	No / missing	No
		Missing
	Yes	Yes
Senior operating surgeon grade	Consultant	Consultant
	Non-consultant	Post CCT fellow
		Specialty trainee
		SAS doctor
		Research/clinical fellow
		Core trainee
		Other
Operative approach	Open	Open
		Laparoscopic converted
	Minimal access	Laparoscopic
		Laparoscopic assisted
Postoperative care level	Ward	Ward
	HDU or ITU	Level 2
		Level 3

ASA – American Society of Anesthesiology score; CT – computed tomography scan; CCT – certificate of completion of training; SAS – specialty and associate specialist doctors.

Supplementary table 2. Relationships between independent variables and mortality.

Variable	Modelling function
Age	Linear
Preoperative HR	Linear
Preoperative SBP	2-term square and cube polynomial
Time admission to decision to operate	Linear

HR – heart rate; SBP – systolic blood pressure.
