# Supplementary material for: Short-term outcomes after emergency surgery for complicated peptic ulcer disease from the UK National Emergency Laparotomy Audit: a cohort study
Source: BMJ Open. 2018 Aug 20;8(8):e023721. doi: 10.1136/bmjopen-2018-023721 (PMC6104767; doi:10.1136/bmjopen-2018-023721)
Supplement: Author's manuscript [file bmjopen-2018-023721.draft_revisions.pdf]

BMJ Open

BMJ Open is committed to open peer review. As part of this commitment we make the peer review history of every article we publish publicly available.

When an article is published we post the peer reviewers' comments and the authors' responses online. We also post the versions of the paper that were used during peer review. These are the versions that the peer review comments apply to.

The versions of the paper that follow are the versions that were submitted during the peer review process. They are not the versions of record or the final published versions. They should not be cited or distributed as the published version of this manuscript.

BMJ Open is an open access journal and the full, final, typeset and author-corrected version of record of the manuscript is available on our site with no access controls, subscription charges or pay-per-view fees (http://bmjopen.bmj.com).

If you have any questions on BMJ Open's open peer review process please email info.bmjopen@bmj.com

BMJ Open

Short-term outcomes after emergency surgery for complicated peptic ulcer disease from the National Emergency Laparotomy Audit

Journal:	*BMJ Open*
Manuscript ID	bmjopen-2018-023721
Article Type:	Research
Date Submitted by the Author:	24-Apr-2018
Complete List of Authors:	Byrne, Benjamin; University of Bristol, Centre for Surgical Research, Population Health Sciences; University Hospitals Bristol NHS Foundation Trust, Department of Surgery<br>Bassett, Michael; Royal College of Anaesthetists, National Emergency Laparotomy Audit; Manchester University NHS Foundation Trust, Department of Anaesthesia<br>rogers, chris; University of Bristol, Clinical Trials and Evaluation Unit<br>Anderson, Iain; Royal College of Anaesthetists, National Emergency Laparotomy Audit; Salford Royal NHS Foundation Trust, Department of Surgery<br>Beckingham, Ian; Nottingham University Hospitals NHS Trust, Department of Surgery; Royal College of Surgeons of England, Association of Upper Gastrointestinal Surgeons<br>Blazeby, Jane; University of Bristol, Centre for Surgical Research, Population Health Sciences; University Hospitals Bristol NHS Foundation Trust, Department of Surgery
Keywords:	EPIDEMIOLOGY, Gastroduodenal disease < GASTROENTEROLOGY, Clinical audit < HEALTH SERVICES ADMINISTRATION & MANAGEMENT, Quality in health care < HEALTH SERVICES ADMINISTRATION & MANAGEMENT, SURGERY

SCHOLARONE™
Manuscripts

Short-term outcomes after emergency surgery for complicated peptic ulcer disease from the National Emergency Laparotomy Audit.

B E Byrne[1], M Bassett[2,3], C A Rogers[4], I D Anderson[2,5], I Beckingham[6,7], J M Blazeby[1,8] for the National Emergency Laparotomy Project Team and on behalf of the Association of Upper Gastrointestinal Surgeons

[1]Centre for Surgical Research, Population Health Sciences, Bristol Medical School, University of Bristol, Bristol, UK.

[2]National Emergency Laparotomy Audit, The Royal College of Anaesthetists, London, UK.

[3]Department of Anaesthesia, Manchester University NHS Foundation Trust, Manchester, UK.

[4]Clinical Trials and Evaluation Unit, Bristol Medical School, University of Bristol, Bristol, UK.

[5]Department of Surgery, Salford Royal NHS Foundation Trust, Salford, UK.

[6]Department of Surgery, Nottingham University Hospitals NHS Trust, Nottingham, UK.

[7]Association of Upper Gastrointestinal Surgeons, The Royal College of Surgeons of England, London, UK.

[8]Department of Surgery, University Hospitals Bristol NHS Foundation Trust, Bristol, UK.

Correspondence: B E Byrne

Address: Centre for Surgical Research, Population Health Sciences, Bristol Medical School, University of Bristol, Canynge Hall, 39 Whatley Road, Clifton, Bristol BS8 2PS, UK.

Email: benbyrne@doctors.org.uk.

Telephone: 0117 928 7279

Word count: 3056

For peer review only

ABSTRACT

Objectives: This study used national audit data to describe current management and outcomes of patients undergoing surgery for complications of peptic ulcer disease, including perforation and bleeding. It was also planned to explore factors associated with fatal outcome after surgery for perforated ulcers. These analyses were designed to provide a thorough understanding of current practice, and identify potentially modifiable factors associated with outcome as targets for future quality improvement.

Design: National cohort study using National Emergency Laparotomy Audit (NELA) data.

Setting: English and Welsh hospitals within the National Health Service.

Participants: Adult patients admitted as an emergency with perforated or bleeding peptic ulcer disease between December 2013 and November 2015.

Interventions: Laparotomy for bleeding or perforated peptic ulcer.

Primary and secondary outcome measures: The primary outcome was 60-day in-hospital mortality. Secondary outcomes included length of postoperative stay, readmission and reoperation rate.

Results: 2444 and 382 procedures were performed for perforated and bleeding ulcers, respectively. In-hospital 60-day mortality rates were 287/2444 (11.7%, 95% confidence intervals (CI) 10.5–13.1%) for perforations, and 68/382 (17.8%, CI 14.1–22.0%) for bleeding. Median (interquartile range) 2-year institutional volume was 12 (7-17) and 2 (1-3) for perforation and bleeding, respectively. Age, American Society of Anesthesiology score and pre-operative systolic blood pressure were associated

For peer review only - http://bmjopen.bmj.com/site/about/guidelines.xhtml

with mortality, with no association with time from admission to operation, surgeon grade or operative approach.

Conclusions: Patients undergoing surgery for complicated PUD face a high 60-day mortality risk. Exploratory analyses suggested fatal outcome was primarily associated with patient rather than provider care factors. Therefore, it may be challenging to reduce mortality rates further. NELA data provides important benchmarking for patient consent, and has highlighted low institutional volume and high mortality rates after surgery for bleeding peptic ulcers as a target for future research and improvement.

For peer review only

ARTICLE SUMMARY

Strengths and limitations of this study

- This multicentre study examined usual clinical practice across a large number of hospitals in the National Health Service in England and Wales, representing the largest study of complicated peptic ulcer disease yet reported in the United Kingdom.
- Structured data was collected prospectively, mitigating against bias associated with retrospective study design.
- However, case ascertainment within the entire National Emergency Laparotomy Audit patient cohort, the main dataset from which the current study data was extracted, was 83% and missing data may have introduced unknown biases into the study.

For peer review only

INTRODUCTION

Surgical treatment of peptic ulcer disease (PUD) has changed markedly over recent years. Overall operative intervention has declined, with a substantial fall in elective procedures such as gastric resection, vagotomy and pyloroplasty[1–3]. However, there remains clinical need for surgical treatment for complications of PUD, with emergency procedures now representing the principal role for operative intervention in this condition[4]. Earlier studies and nationwide audits show postoperative mortality following emergency surgery for perforated or bleeding ulcers to range from 9.1% to 26.5% although data from contemporary UK practice is lacking[2,5–8].

The National Emergency Laparotomy Audit (NELA) is a mandatory audit that captures rich data about the care of patients undergoing a range of emergency bowel operations in England and Wales. NELA was established in 2012 and is run by the Royal College of Anaesthetists, in collaboration with the Clinical Effectiveness Unit at the Royal College of Surgeons of England. The audit aims to improve the quality of care for patients undergoing emergency laparotomy, by collecting information on patients, the processes of care they receive and their short-term outcomes. These data are fed back locally, as well as being analysed nationally, and compared against accepted audit standards. To date, there have been three audit reports, most recently documenting a 30-day post-operative mortality rate of 10.6% (95% confidence intervals (CI) 10.2% – 11.0%) and a median length of stay of 11 days across the range of patients and conditions included[9–11].

The present study aimed to use NELA data to identify patients undergoing surgery for perforated or bleeding PUD, to describe the latest management and short-term outcomes for these patients. The study also explored factors that may be associated with mortality after surgery for perforated PUD. A thorough appraisal of current practice is critical for benchmarking performance, and appropriately directing future research and quality improvement.

For peer review only - http://bmjopen.bmj.com/site/about/guidelines.xhtml

For peer review only

METHODS

The NELA database contains information collected at the level of individual operations for patients, covering details of the admission, pre-operative management and risk-stratification, intra-operative details, post-operative risk and patient outcomes. From this database, patients aged 18 years or over undergoing 'Peptic ulcer – suture or repair of perforation' or 'Peptic ulcer – oversew of bleed' as their first, main surgical procedure after admission, between 1st December 2013 and 30th November 2015, were selected for inclusion. Re-operations and patients undergoing 'Gastric surgery – other', which is likely to have included formal surgical resection, were excluded. Data for the first and second years of the study were extracted on 1st February in 2015 and 2016, respectively. Data on age, sex, American Society of Anesthesiologists (ASA) score, pre-operative heart rate (HR), pre-operative systolic blood pressure (SBP), pre-operative predicted mortality (P-POSSUM)[12] and morbidity (POSSUM) were extracted[13]. NELA specifies recording of HR and SBP values closest to the time of booking the patient for theatre. Pre-operative care details, including the use of Computed Tomography (CT), time from admission to operation, time from admission to decision to operate, time from decision to operate to operation, and time from admission to antibiotics were also recorded. Information on the grade of most senior operating surgeon and surgical approach (open or minimal access), as well as intra-operative findings (extent and type of peritoneal contamination, see supplementary table 1), were examined, along with the immediate postoperative level of care (ward, level 2 or high dependency unit (HDU), and level 3 or intensive care unit (ICU)) and hospital procedure volume. The following outcomes were examined: total length of stay in an enhanced care setting (HDU or ITU); total postoperative length of stay; return to theatre; and in-hospital death within 60 days of the primary operative procedure.

Statistical analysis

For peer review only - http://bmjopen.bmj.com/site/about/guidelines.xhtml

Patients were grouped according to presentation with perforation or bleeding secondary to PUD. Continuous data were described using mean and standard deviation or median and interquartile range if skewed, and category data were summarised as number and percentage. Length of stay was summarised using survival methods with deaths prior to discharge treated as censored observations.

For patients with perforated PUD, associations between patient, care and operative factors and mortality were examined using multilevel logistic regression, with hospital fitted as random effect. For this analysis alone, only patients undergoing surgery for perforation within 48 hours of admission to hospital were included. This was designed to exclude patients who developed a perforation during their admission, and patients undergoing surgery after an unsuccessful period of non-operative management. Such patients may represent a different population with a different risk profile. For example, severely comorbid patients with mild clinical signs may preferentially be selected for initial non-operative management. The following variables were selected for exploration by consensus within the working group before analysis: age; sex; ASA; HR; SBP; pre-operative CT; time from admission to operation; grade of senior operating surgeon; operative approach; peritoneal contamination type; peritoneal contamination extent; and post-operative care level. Variables with many categories were grouped for analysis (see supplementary table 1). Fractional polynomials were used to describe the relationship between continuous variables and mortality (supplementary table 2). Data that was clearly incorrect was recoded as missing. The analysis was restricted to cases with complete data for the variables of interest. All variables were included in the model and not selected based on statistical significance. All analysis was conducted using Stata 14.0 (StataCorp, College Station, TX).

Ethics

For peer review only - http://bmjopen.bmj.com/site/about/guidelines.xhtml
For peer review only

NELA has approval from the Health Research Authority's Confidentiality Advisory Group for 'Use of Patient Identifiable Information without Consent' (Section 251 of NHS Act 2006 and Health Service (Control of Patient Information) Regulations 2002). The present analysis was performed under NELA's remit to understand and inform the delivery of care to patients undergoing emergency laparotomy. The data extract included anonymised patient level data. Therefore, further approval by a research ethics committee was not required. Participating Trusts follow local governance arrangements for audit registration. Patient data are uploaded via an encrypted website to a secure server. Access is carefully restricted and data used in accordance with the Caldicott principles[14].

Patient involvement

Patients were not involved in any aspect of the design or conduct of this study.

For peer review only

For peer review only - http://bmjopen.bmj.com/site/about/guidelines.xhtml

RESULTS

Patient characteristics and hospital volume

During the study period, 43 321 emergency laparotomies were identified at 192 hospitals in England and Wales. Data on 2444 (5.5%) perforated peptic ulcers and 382 (0.9%) bleeding ulcers were retrieved from 186 (96.9%) contributing hospitals. Patient characteristics are shown in table 1. Over the two-year period, the median number of cases per hospital was 12 (IQR 7-17) and 2 (IQR 1-3) for perforated and bleeding ulcers, respectively.

Table 1. Pre-operative details of patients undergoing surgery for perforation or bleeding.

		Perforation		Bleed		All PUD	
		n=2444	(%)	n=382	(%)	n=2826	(%)
Age (years)		57.8	(19.4)	65.0	(16.3)	58.8	(19.2)
Sex	Male	1450	(59.3)	240	(62.8)	1690	(59.8)
ASA	1	569	(23.3)	30	(7.9)	599	(21.2)
	2	738	(30.2)	64	(16.8)	802	(28.4)
	3	611	(25.0)	98	(25.7)	709	(25.1)
	4	461	(18.9)	158	(41.4)	619	(21.9)
	5	65	(2.7)	32	(8.4)	97	(3.4)
Pre-op heart rate	<80	449	(18.6)	47	(12.5)	496	(17.8)
	80-99	928	(38.4)	140	(37.1)	1068	(38.2)
	100-119	704	(29.1)	109	(28.9)	813	(29.1)
	120-139	267	(11.0)	65	(17.2)	332	(11.9)
	≥140	69	(2.9)	16	(4.2)	85	(3.0)
Pre-op systolic blood pressure	<80	63	(2.6)	37	(11.6)	100	(3.6)
	80-99	260	(10.8)	96	(30.1)	356	(12.8)
	100-119	670	(27.8)	118	(37.0)	788	(28.3)
	120-139	831	(34.5)	68	(21.3)	899	(32.3)
	140-159	429	(17.8)	43	(11.4)	472	(16.9)
	≥160	157	(6.5)	15	(4.0)	172	(6.2)
Predicted mortality (P-POSSUM)	<5%	935	(38.3)	49	(12.8)	984	(34.8)
	5-9%	416	(17.0)	37	(9.7)	453	(16.0)
	10-24%	445	(18.2)	74	(19.4)	519	(18.4)
	25-49%	292	(11.9)	83	(21.7)	375	(13.3)
	≥50%	356	(14.6)	139	(36.4)	495	(17.5)
Predicted morbidity (POSSUM)	<25%	54	(2.2)	2	(0.5)	56	(2.0)
	25-49%	385	(15.8)	16	(4.2)	401	(14.2)
	50-74%	747	(30.6)	53	(13.9)	800	(28.3)
	≥75%	1258	(51.5)	311	(81.4)	1569	(55.5)

Age provided as mean (standard deviation); PUD – peptic ulcer disease; ASA – American Society of Anesthesiology score; P-POSSUM – Portsmouth-POSSUM; POSSUM – Physiological and Operative Severity Score for the enUmeration of Mortality and morbidity.

For peer review only - http://bmjopen.bmj.com/site/about/guidelines.xhtml

For peer review only

Pre-operative care

Pre-operative imaging differed according to diagnosis, with the majority (1792 / 2444, 73.3%) of patients undergoing treatment for perforated ulcers receiving a pre-operative CT scan (table 2), compared with 101 / 382 (26.4%) of patients with a bleeding ulcer.

Table 2. Details of pre-operative care of patients undergoing surgery for perforation or bleeding.

		Perforation		Bleed		All PUD	
		n=2444	(%)	n=382	(%)	n=2826	(%)
Pre-operative CT	Yes	1792	(74.1)	101	(26.8)	1893	(67.7)
	No	626	(25.9)	276	(73.2)	902	(32.3)
Time (hours)	Admission to operation	8.8	(5.3-18.9)	30.4	(9.4-107.8)	9.7	(5.5-23.4)
	Admission to decision to operate	6.0	(3.1-14.6)	29.3	(7.5-119.3)	6.5	(3.3-19.4)
	Decision to operate to operation	2.0	(1.2-3.4)	1.1	(0.5-2.1)	1.9	(1.1-3.2)
	Admission to first antibiotics	4.6	(2.1-10.1)	11.8	(3.8-47.8)	5.0	(2.3-11.9)

Timing data provided as median (interquartile range); PUD – peptic ulcer disease; CT – computed tomography scan.

Median interval from admission to surgery was 8.8 hours (IQR 5.3 – 18.9) in patients with a perforated ulcer, and 30.4 hours (IQR 9.4 – 107.8) in those with a bleeding ulcer. Median interval from decision to operate to surgery was 2.0 (IQR 1.2 – 3.4) and 1.1 (IQR 0.5 – 2.1) hours in patients with perforations and bleeding ulcers, respectively. Patients admitted with a perforated ulcer received their first dose of antibiotics at a median of 4.6 (IQR 2.1 – 10.1; data recorded for 2085 / 2444 (85.3%) of patients) hours after admission, and those undergoing surgery for bleeding ulcers received antibiotics at a median of 11.8 hours (IQR 3.8 – 47.8; data for 273 / 382 (71.5%) of patients).

Operative details and postoperative care level

For peer review only - http://bmjopen.bmj.com/site/about/guidelines.xhtml

Consultants were recorded as the senior surgeon in the majority of cases and most surgery was performed via the open approach (table 3). However, 489 / 2 444 (20.0%) patients underwent some form of laparoscopic surgery for their perforated ulcer, with 320 (13.1%) procedures completed laparoscopically. The nature and extent of peritoneal contamination differed according to the clinical problem. The majority of patients with perforated ulcers had significant contamination affecting multiple quadrants.

Table 3. Operative details and postoperative destination for patients undergoing surgery for perforation or bleeding.

			Perforation		Bleed		All PUD	
			n=2444	(%)	n=382	(%)	n=2826	(%)
Operation	Senior surgeon	Consultant	1763	(72.1)	347	(90.1)	2110	(74.7)
		Specialty trainee	453	(18.5)	27	(7.1)	480	(17.0)
		Other	228	(9.3)	8	(2.1)	236	(8.4)
	Approach	Open	1955	(80.0)	367	(96.1)	2322	(82.2)
		Laparoscopic (including assisted)	320	(13.1)	10	(2.6)	330	(11.7)
		Laparoscopic converted	169	(6.9)	5	(1.3)	174	(6.2)
	Contamination type	None / minimal	425	(17.4)	250	(65.4)	675	(23.9)
		Significant	2019	(82.6)	132	(34.6)	2151	(76.1)
	Contamination extent	None / single quadrant	753	(30.8)	319	(83.5)	1072	(37.9)
		Multiple quadrants	1691	(69.2)	63	(16.5)	1754	(62.1)
Postoperative care level		Ward (level 1)	1015	(41.5)	68	(17.8)	1083	(38.4)
		HDU (level 2)	652	(26.7)	99	(25.9)	751	(26.6)
		ITU (level 3)	774	(31.7)	212	(55.5)	986	(35.0)

PUD – peptic ulcer disease; HDU – high dependency unit; ITU – intensive therapy unit.

Most patients who underwent surgery to repair a perforated ulcer (1426 / 2444, 58.3%) were transferred to a HDU or ITU environment, with the remainder being transferred to a ward. A much higher proportion of patients operated on for bleeding went to HDU or ITU after surgery (311 / 382, 81.4%).

Outcomes

For peer review only - http://bmjopen.bmj.com/site/about/guidelines.xhtml

Among patients transferred to HDU or ITU, the median postoperative stay in an enhanced care environment was 4 days for both groups (table 4). The median total postoperative stay was 8.4 days for patients treated for a perforated ulcer, compared with a longer median stay of 15.0 days for bleeding ulcers. The rate of return to theatre was lower among patients operated on for perforation (136 / 2 444, 5.6%) than after surgery for bleeding (36 / 382, 9.4%). In each group, three patients died in theatre. The overall, 60-day in-hospital mortality was 287 / 2 444 (11.7%, 95% CI 10.5% – 13.1%) after surgery for a perforated ulcer, and 68 / 382 (17.8%, 95% CI 14.1% – 22.0%) after oversew of a bleeding ulcer.

Table 4. Outcomes of patients undergoing surgery for perforation or bleeding.

		Perforation n=2 444	(%)	Bleed n=382	(%)	All PUD n=2826	(%)
Length of stay (days)	HDU / ITU	4.0	(2.0-7.0)	4.0	(2.0-8.0)	4.0	(2.0-7.0)
	Total	8.4	(5.2-18.4)	15.0	(7.5-29.0)	9.2	(5.4-20.0)
Return to theatre		136	(5.6)	36	(9.4)	172	(6.1)
Mortality in-hospital within 60 days		287	(11.7)	68	(17.8)	355	(12.6)
	(Died in theatre)	3	(0.1)	3	(0.8)	6	(0.2)

Length of stay provided as median (interquartile range). PUD – peptic ulcer disease; HDU – high dependency unit; ITU – intensive therapy unit.

Exploratory analysis

Of 2 327 patients where time from admission to operation was recorded, 2 231 (96.1%) underwent surgery for perforation in the first 48 hours after admission. Complete data for regression analysis were available for 2 162 (96.7%) of these patients. Variables identified as significantly associated with in-hospital 60-day mortality (after accounting for other variables) were age, ASA, pre-operative SBP and post-operative care level (table 5). For each increasing year of age, the risk of death rose by 5.0% (95% CI 3.5% – 6.5%), meaning that an increase of 10 years of age was associated with increased risk of death of 63.3% (95% CI 41.6% – 88.4%). An ASA score of 4 or 5 was associated with a markedly elevated risk of fatal outcome compared to an ASA of 1. There was also an increased risk

For peer review only - http://bmjopen.bmj.com/site/about/guidelines.xhtml

of death for patients going to HDU or ITU compared to those transferred directly to a ward. The association between pre-operative SBP and postoperative mortality was non-linear (illustrated in fig 1). Extremes of low or high SBP were associated with increased risk of death. There was no statistically significant association observed between patient sex, pre-operative HR, use of pre-operative CT, time from admission to operation, operating surgeon, operative approach, intra-operative contamination type or extent and subsequent in-hospital mortality.

Table 5. Multilevel logistic regression results, examining factors associated with 60-day in-hospital mortality after surgery for perforation; analysis restricted to patients undergoing surgery within 48 hours of admission.

		Odds ratio	95% confidence intervals Lower	Upper	*p*-value
Age (per year)		1.05	1.04	1.07	<0.001
Sex	Male	1.00			
	Female	1.00	0.70	1.42	0.999
ASA	1	1.00			<0.001
	2	0.77	0.26	2.26	
	3	2.10	0.77	5.76	
	4 & 5	7.19	2.62	19.73	
Pre-op heart rate (per 10 bpm)		1.03	0.95	1.11	0.529
Pre-op systolic blood pressure*					<0.001
Pre-op CT	No	1.00			
	Yes	1.41	0.90	2.22	0.133
Time from admission to operation (per hour)		1.01	0.99	1.02	0.392
Operating surgeon	Consultant	1.00			
	Non-consultant	0.90	0.57	1.40	0.633
Operative approach	Open	1.00			
	Laparoscopic (inc. assisted)	0.78	0.40	1.50	0.459
Contamination type	None / minimal	1.00			
	Significant	0.88	0.49	1.58	0.660
Contamination extent	None / single quadrant	1.00			
	Multiple quadrants	1.14	0.70	1.84	0.605
Postoperative destination	Ward	1.00			
	HDU or ITU	2.22	1.20	4.11	0.011

* - non-linear relationship; ASA – American Society of Anesthesiology score; CT – computed tomography scan; HDU – high dependency unit; ITU – intensive therapy unit.

For peer review only - http://bmjopen.bmj.com/site/about/guidelines.xhtml
For peer review only

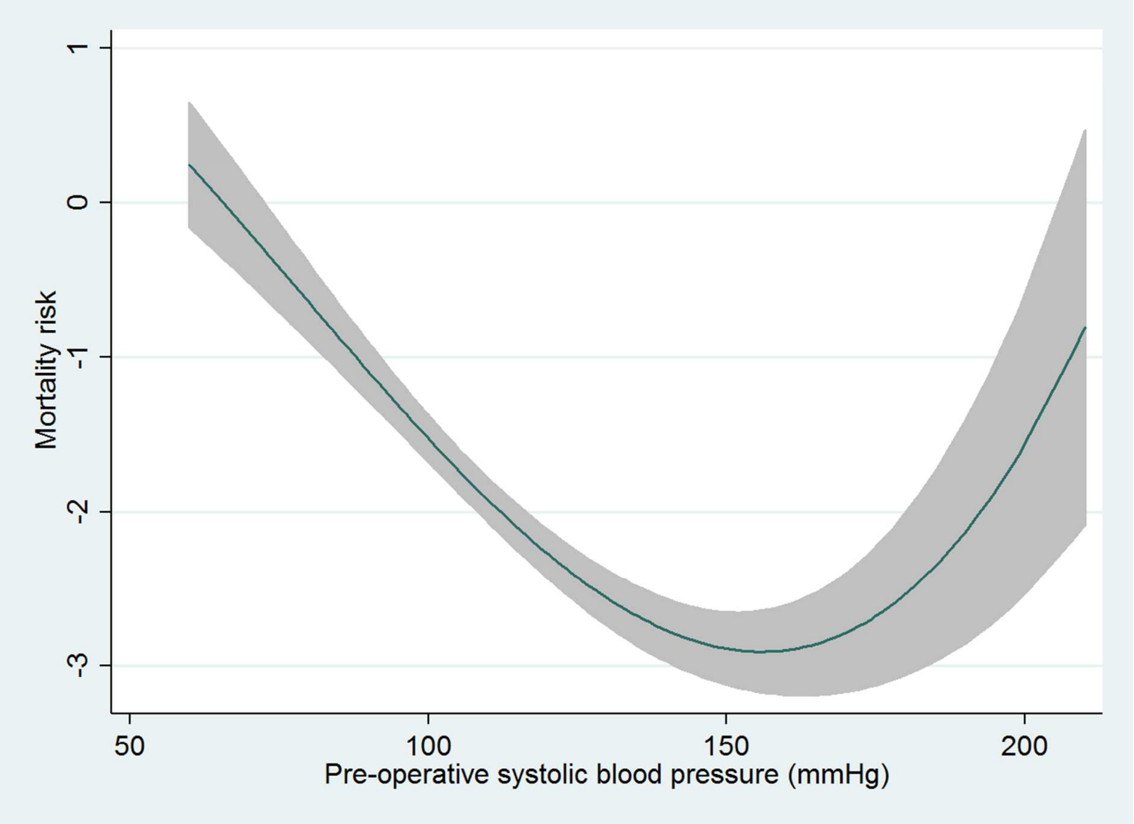

Fig 1. Illustration of non-linear relationship between pre-operative systolic blood pressure and 60-day in-hospital mortality.

For peer review only - http://bmjopen.bmj.com/site/about/guidelines.xhtml

DISCUSSION

This is the first national study in the United Kingdom of complicated peptic ulcer disease requiring emergency surgery. Using two years of NELA data, we identified 2444 and 382 patients from 186 English and Welsh hospitals undergoing surgery for perforated or bleeding PUD. The post-operative in-hospital 60-day mortality rates were 11.7% and 17.8%, respectively. Mortality after repair of perforated ulcer was primarily associated with patient factors, rather than more easily modifiable aspects of the care provided. This may make it difficult to reduce mortality rates further. Average institutional surgical volume for bleeding ulcers was very low, and these patients had the highest mortality risk, highlighting the challenge and urgent need for further work to understand and improve outcomes in this group.

While mortality rates among the included patients were high, the reported results compare favourably with data from other research. Recent European studies have reported 90-day mortality rates from 19.2% - 29.8% after surgery for perforated PUD[5,15]. Among patients undergoing surgery for bleeding peptic ulcers, 30-day mortality rates were higher, ranging from 23.7% to 25.6%[7,16], with previous UK research revealing a 30% (CI 22% – 38%) post-operative mortality rate[17]. A recent large US study using American College of Surgeons National Surgical Quality Improvement Program data demonstrated similar rates to those observed in this study with 12.1% (CI 10.8% - 13.5%) and 18.6% (CI 15.9% - 21.5%) 30-day mortality after surgery for perforation and bleeding PUD respectively.

The exploratory analysis of factors associated with mortality after repair of perforation generated new, unexpected findings. The significant associations between age, ASA and preoperative SBP and post-operative mortality are unsurprising and agree with previous research[18]. However, the lack of association with time from admission to surgery disagrees with the published literature. For

For peer review only - http://bmjopen.bmj.com/site/about/guidelines.xhtml

example, Buck et al reported that after adjusting for prognostic variables, each hour of surgical delay during the first 24 hours of admission was associated with a 2.4% (CI 1.1 – 3.7%) decrease in the probability of survival[19]. However, that study used less nuanced modelling of continuous variables such as age or shock, which were reduced to dichotomous variables. In addition, they did not describe any exclusion criteria based on time from admission to surgery, raising the possibility that their cohort included patients undergoing surgery after failed conservative management, and those developing a perforation during their hospital admission. The present lack of association between time from admission to operation has important clinical implications, especially when considered alongside the significant association between pre-operative SBP and mortality. Together, these findings may be interpreted to suggest that a short delay in transfer to the operating theatre for appropriate investigations or fluid optimisation is unlikely to compromise survival outcomes. Indeed, if it is possible to improve a patient's physiological condition and SBP, this has potential to reduce mortality rates. However, caution must be exercised in this interpretation, and future research should explore this finding in more detail.

This study found that 13.1% of patients underwent surgical repair of their perforated ulcer via a laparoscopic approach. A further 6.9% were converted from laparoscopic to open. Although the reasons for conversion were not recorded, it is possible that some patients underwent an initial diagnostic laparoscopy before proceeding directly to open repair once the a diagnosis was made. A smaller Danish study of 726 patients undergoing surgery for perforated PUD reported a laparoscopy rate of 32.8%, with 24.5% converted from laparoscopic to open[20]. The lower rate in the present study may represent under-reporting, as, anecdotally, some clinicians may not have considered a laparoscopic suture repair eligible for a laparotomy audit. Future comparison with Hospital Episode Statistics administrative data, available for all NHS activity, could test this hypothesis. Alternatively, the lower rate may be a true reflection of practice, and lack of skills or confidence in performing laparoscopic repair. The lack of association between operative approach and outcome may suggest

For peer review only - http://bmjopen.bmj.com/site/about/guidelines.xhtml

For peer review only

that patients are being treated laparoscopically on the availability of appropriately skilled surgeons, rather than through careful case selection or 'picking winners'. Further and more detailed analysis is warranted.

This study has not defined clear ways to improve the survival of the patients included. It has, however, identified aspects of the care provided that were not associated with mortality, suggesting that these should not be the primary focus for immediate quality improvement. The results provide no evidence that more rapid transfer to the operating theatre, greater consultant presence or adoption of minimal access techniques would improve survival rates. Preoperative SBP is potentially modifiable and may be an appropriate target for future research and quality improvement. Selection of patients for postoperative care in the HDU or ITU environment, and other variables not analysed due to missing data, such as time from admission to antibiotics, should also be investigated further.

The results highlight the low institutional volume for the included procedures, particularly for bleeding PUD requiring surgery. Endoscopy is the first line investigation and treatment for upper gastrointestinal bleeding, and previous large studies have demonstrated the high success rates of endoscopic therapy for bleeding ulcers, with surgery required in 1.9-5.4% of cases[5,16,17]. National guidance in the UK suggests interventional radiology and embolisation should be offered as second-line treatment[21], but few hospitals have 24/7 access to this service. When requiring surgery, these patients are high risk, as reflected in their ASA scores, with associated high levels of senior involvement in theatre. Low procedure volumes make it difficult to develop expertise managing these patients, which in turn may make it difficult to improve outcomes. In several surgical specialties, higher procedure volume has been associated with improved outcomes[22–24]. However, it is not clear whether such a volume-outcome relationship exists for emergency surgery for bleeding peptic ulcers. It would likely be difficult to centralise secondary treatment required for

For peer review only - http://bmjopen.bmj.com/site/about/guidelines.xhtml

For peer review only

an unstable patient. Further research, using quantitative databases and case-studies in different centres, may determine future strategies to improve care for these patients.

This study has several strengths, as a nationwide prospective audit. However, there are limitations. Whilst participation is mandatory, case ascertainment was estimated at 83% in the first two years of the audit[9,10]. No eligible procedures were identified in 6 (3.1%) of hospitals participating in NELA. Research in other areas has found that voluntary clinical databases typically demonstrate a lower mortality rate than population-based administrative data[25,26]. Therefore, it is possible that mortality rates across the country are higher than observed, further highlighting the need for more work in this area. In addition, deaths after discharge, or during the index admission but more than 60 days after the operative procedure, were not included. Another limitation is possible variation in coding of information, which depends upon how different observers interpret the terms. For example, coding of contamination in bleeding ulcers may have reflected existing contamination, or it may have reflected contamination due to the enterotomy required to visualise and treat the bleeding ulcer. It is not possible to retrospectively check the accuracy of such data, which must be taken at face value. While data completeness was satisfactory for the analysis presented, it is not possible to determine whether missing data introduced systematic bias. The extent of missing data precluded exploratory analysis of further variables of interest, such as time from admission to antibiotics, and time from admission to decision to operate. While the results may be cautiously generalised to similar populations and healthcare systems, differences in care organisation may limit broad applicability.

In summary, this national study has demonstrated mortality rates within the NHS in England and Wales that compare favourably with previously published international results. The overall rate of mortality, however, remains high. Exploratory analysis suggested fatal outcome after surgery for perforation was primarily associated with patient factors rather than the care provided, and this may

For peer review only - http://bmjopen.bmj.com/site/about/guidelines.xhtml

make further improvement difficult. As NELA accrues more data over the remaining years of the project, it may be feasible to explore the association between other, modifiable care factors, such as time to antibiotics, and clinical outcomes and this could aid further research. Surgical management of bleeding PUD represents an area of practice with very low volume and high postoperative mortality that mandates further investigation. Centralisation may be considered, though this could be difficult due to the acuity of patients requiring surgery in this setting. Research using future audit data may guide quality improvement efforts, to benefit patients requiring surgery for complications of PUD.

For peer review only

For peer review only - http://bmjopen.bmj.com/site/about/guidelines.xhtml

Acknowledgements

The authors would like to thank the members of the NELA Project Team during the data collection period of this study: Mr Martin Cripps, Professor David Cromwell, Dr Emma Davies, Ms Sharon Drake, Dr Mike Galsworthy, Professor Mike Grocott, Dr Angela Kuryba, Mr Jose Lourtie, Dr Ramani Moonesinghe, Dr Dave Murray, Dr Matt Oliver, Mr Dimitri Papadimitriou, Dr Carol Peden, Dr Kate Walker.

Funding

B E Byrne is an academic clinical fellow in surgery supported by the National Institute for Health Research (NIHR). J M Blazeby is a NIHR Senior Investigator. This work was undertaken with the support of the MRC ConDuCT-II (Collaboration and innovation for Difficult and Complex randomised controlled Trials In Invasive procedures) Hub for Trials Methodology Research (MR/K025643/1). The study was also supported by the NIHR Biomedical Research Centre at the University Hospitals Bristol NHS Foundation Trust and the University of Bristol. The views expressed are those of the authors and not necessarily those of the UK National Health Service, National Institute for Health Research, or the Department of Health. The National Emergency Laparotomy Audit is commissioned by the Healthcare Quality Improvement Partnership as part of the National Clinical Audit Programme on behalf of NHS England and the Welsh Government. The funders had no role in study design, data collection and analysis, decision to publish, or preparation of the manuscript.

Competing interests

The authors have declared that no competing interests exist.

For peer review only

For peer review only - http://bmjopen.bmj.com/site/about/guidelines.xhtml

Contributions

IDA, IB and JMB conceived the study. MB and IDA acquired the data. BEB, MB and CAR analysed the data. All authors interpreted the data. BEB drafted the manuscript. MB, CAR, IDA, IB and JMB critically revised the manuscript for important intellectual content. All authors approved the final version for publication.

Data sharing

No additional data available.

Previous presentation

Parts of this work have been presented at the Association of Surgeons of Great Britain and Ireland International Surgical Congress, Glasgow, May 2017, and at the Association of Upper Gastro-Intestinal Surgeons 20th Annual Scientific Meeting, Cork, September 2017.

For peer review only

For peer review only - http://bmjopen.bmj.com/site/about/guidelines.xhtml

REFERENCES

1 Bashinskaya B, Nahed B V, Redjal N, *et al.* Trends in peptic ulcer disease and the identification of Helicobacter Pylori as a causative organism: population-based estimates from the US Nationwide Inpatient Sample. *J Glob Infect Dis* 2011;**3**:366–70.

2 Wang YR, Richter JE, Dempsey DT. Trends and outcomes of hospitalizations for peptic ulcer disease in the United States, 1993 to 2006. *Ann Surg* 2010;**251**:51–8. doi:10.1097/SLA.0b013e3181b975b8

3 Bardhan KD, Williamson M, Royston C, *et al.* Admission rates for peptic ulcer in the Trent Region, UK, 1972-2000. Changing pattern, a changing disease? *Dig Liver Dis* 2004;**36**:577–88. doi:10.1016/j.dld.2004.04.007

4 Paimela H, Oksala NKJ, Kivilaakso E. Surgery for peptic ulcer today: a study on the incidence, methods and mortality in surgery for peptic ulcer in Finland between 1987 and 1999. *Dig Surg* 2004;**21**:185–91. doi:10.1159/000079654

5 Lolle I, Møller MH, Rosenstock SJ. Association between ulcer site and outcome in complicated peptic ulcer disease: a Danish nationwide cohort study. *Scand J Gastroenterol* 2016;**51**:1165–71. doi:10.1080/00365521.2016.1190398

6 Lau JY, Sung J, Hill C, *et al.* Systematic review of the epidemiology of complicated peptic ulcer disease: incidence, recurrence, risk factors and mortality. *Digestion* 2011;**84**:102–13. doi:10.1159/000323958

7 Kyaw M, Tse Y, Ang D, *et al.* Embolization versus surgery for peptic ulcer bleeding after failed endoscopic hemostasis: a meta-analysis. *Endosc Int Open* 2014;**2**:E6–14. doi:10.1055/s-0034-1365235

8 Schroder VT, Pappas TN, Vaslef SN, *et al.* Vagotomy/Drainage is superior to local oversew in patients who require emergency surgery for bleeding peptic ulcers. *Ann Surg* 2014;**259**:1111–8. doi:10.1097/SLA.0000000000000386

For peer review only

For peer review only - http://bmjopen.bmj.com/site/about/guidelines.xhtml

9 NELA Project Team. The first patient report of the national emergency laparotomy audit. 2015.

10 NELA Project Team. The second patient report of the National Emergency Laparotomy Audit. 2016. http://www.nela.org.uk/reports (accessed 18 May 2017).

11 NELA Project Team. Third patient report of the National Emergency Laparotomy Audit. 2017.

12 Prytherch DR, Whiteley MS, Higgins B, *et al.* POSSUM and Portsmouth POSSUM for predicting mortality. *Br J Surg* 1998;**85**:1217–20. doi:10.1046/j.1365-2168.1998.00840.x

13 Copeland GP, Jones D, Walters M. POSSUM: a scoring system for surgical audit. *Br J Surg* 1991;**78**:355–60. doi:10.1002/bjs.1800780327

14 NELA Project Team. Information governance procedures for NELA. http://nela.org.uk/download.php/?fn=Information Governance Procedures for NELA.pdf&mime=application/pdf&pureFn=Information Governance Procedures for NELA.pdf (accessed 18 May 2017).

15 Thorsen K, Søreide JA, Søreide K. Long-term mortality in patients operated for perforated peptic ulcer: factors limiting longevity are dominated by older age, comorbidity burden and severe postoperative complications. *World J Surg* 2017;**41**:410–8. doi:10.1007/s00268-016-3747-z

16 Nykänen T, Peltola E, Kylänpää L, *et al.* Bleeding gastric and duodenal ulcers: case-control study comparing angioembolization and surgery. *Scand J Gastroenterol* 2017;**52**:523–30. doi:10.1080/00365521.2017.1288756

17 Hearnshaw SA, Logan RFA, Lowe D, *et al.* Acute upper gastrointestinal bleeding in the UK: patient characteristics, diagnoses and outcomes in the 2007 UK audit. *Gut* 2011;**60**:1327–35. doi:10.1136/gut.2010.228437

18 Møller MH, Adamsen S, Thomsen RW, *et al.* Preoperative prognostic factors for mortality in peptic ulcer perforation: A systematic review. *Scand J Gastroenterol* 2010;**45**:785–805. doi:10.3109/00365521003783320

For peer review only

For peer review only - http://bmjopen.bmj.com/site/about/guidelines.xhtml

19 Buck DL, Vester-Andersen M, Møller MH. Surgical delay is a critical determinant of survival in perforated peptic ulcer. *Br J Surg* 2013;**100**:1045–9. doi:10.1002/bjs.9175

20 Wilhelmsen M, Møller MH, Rosenstock S. Surgical complications after open and laparoscopic surgery for perforated peptic ulcer in a nationwide cohort. *Br J Surg* 2015;**102**:382–7. doi:10.1002/bjs.9753

21 National Institute For Health and Care Excellence. Acute upper gastrointestinal bleeding in over 16s: management. 2012. https://www.nice.org.uk/guidance/cg141/resources/acute-upper-gastrointestinal-bleeding-in-over-16s-management-pdf-35109565796293

22 Birkmeyer JD, Stukel T a, Siewers AE, *et al.* Surgeon volume and operative mortality in the United States. *N Engl J Med* 2003;**349**:2117–27. doi:10.1056/NEJMsa035205

23 Markar SR, Karthikesalingam A, Thrumurthy S, *et al.* Volume-outcome relationship in surgery for esophageal malignancy: systematic review and meta-analysis 2000-2011. *J Gastrointest Surg* 2012;**16**:1055–63. doi:10.1007/s11605-011-1731-3

24 Pieper D, Mathes T, Neugebauer E, *et al.* State of evidence on the relationship between high-volume hospitals and outcomes in surgery: a systematic review of systematic reviews. *J Am Coll Surg* 2013;**216**:1015–25. doi:10.1016/j.jamcollsurg.2012.12.049

25 Almoudaris AM, Burns EM, Bottle A, *et al.* A colorectal perspective on voluntary submission of outcome data to clinical registries. *Br J Surg* 2011;**98**:132–9. doi:10.1002/bjs.7301

26 Aylin P, Lees T, Baker S, *et al.* Descriptive study comparing routine hospital administrative data with the Vascular Society of Great Britain and Ireland's National Vascular Database. *Eur J Vasc Endovasc Surg* 2007;**33**:461–5. doi:10.1016/j.ejvs.2006.10.033

For peer review only

For peer review only - http://bmjopen.bmj.com/site/about/guidelines.xhtml

SUPPLEMENTARY MATERIAL

Supplementary table 1. Grouping of categorical variables for analysis.

Variable	Group	Original values
ASA	1	1
	2	2
	3	3
	4 & 5	4
		5
Peritoneal contamination type	None / minimal	None / serous
		Gas / minimal
	Significant	Pus
		Bile
		Gastro-duodenal contents
		Small bowel contents
		Faeculent fluid
		Faeces
		Blood
Peritoneal contamination extent	None / single quadrant	None
		Single quadrant
	Multiple quadrants	Multiple quadrants
Preoperative CT	No / missing	No
		Missing
	Yes	Yes
Senior operating surgeon grade	Consultant	Consultant
	Non-consultant	Post CCT fellow
		Specialty trainee
		SAS doctor
		Research/clinical fellow
		Core trainee
		Other
Operative approach	Open	Open
		Laparoscopic converted
	Minimal access	Laparoscopic
		Laparoscopic assisted
Postoperative care level	Ward	Ward
	HDU or ITU	Level 2
		Level 3

ASA – American Society of Anesthesiology score; CT – computed tomography scan; CCT – certificate of completion of training; SAS – specialty and associate specialist doctors.

For peer review only

Supplementary table 2. Relationships between independent variables and mortality.

Variable	Modelling function
Age	Linear
Preoperative HR	Linear
Preoperative SBP	2-term square and cube polynomial
Time admission to decision to operate	Linear

HR – heart rate; SBP – systolic blood pressure.

For peer review only

STROBE 2007 (v4) Statement—Checklist of items that should be included in reports of *cohort studies*

Section/Topic	Item #	Recommendation	Reported on page #
**Title and abstract**	1	(*a*) Indicate the study's design with a commonly used term in the title or the abstract	1
		(*b*) Provide in the abstract an informative and balanced summary of what was done and what was found	2-3
**Introduction**			
Background/rationale	2	Explain the scientific background and rationale for the investigation being reported	5
Objectives	3	State specific objectives, including any prespecified hypotheses	5
**Methods**			
Study design	4	Present key elements of study design early in the paper	6
Setting	5	Describe the setting, locations, and relevant dates, including periods of recruitment, exposure, follow-up, and data collection	6
Participants	6	(*a*) Give the eligibility criteria, and the sources and methods of selection of participants. Describe methods of follow-up	6
		(*b*) For matched studies, give matching criteria and number of exposed and unexposed	NA
Variables	7	Clearly define all outcomes, exposures, predictors, potential confounders, and effect modifiers. Give diagnostic criteria, if applicable	6
Data sources/ measurement	8*	For each variable of interest, give sources of data and details of methods of assessment (measurement). Describe comparability of assessment methods if there is more than one group	*6*
Bias	9	Describe any efforts to address potential sources of bias	NA
Study size	10	Explain how the study size was arrived at	NA
Quantitative variables	11	Explain how quantitative variables were handled in the analyses. If applicable, describe which groupings were chosen and why	7
Statistical methods	12	(*a*) Describe all statistical methods, including those used to control for confounding	7
		(*b*) Describe any methods used to examine subgroups and interactions	7
		(*c*) Explain how missing data were addressed	7
		(*d*) If applicable, explain how loss to follow-up was addressed	NA
		(*e*) Describe any sensitivity analyses	NA
**Results**			

For peer review only

Participants	13*	(a) Report numbers of individuals at each stage of study—eg numbers potentially eligible, examined for eligibility, confirmed eligible, included in the study, completing follow-up, and analysed	9
		(b) Give reasons for non-participation at each stage	NA
		(c) Consider use of a flow diagram	NA
Descriptive data	14*	(a) Give characteristics of study participants (eg demographic, clinical, social) and information on exposures and potential confounders	9
		(b) Indicate number of participants with missing data for each variable of interest	10-11
		(c) Summarise follow-up time (eg, average and total amount)	12
Outcome data	15*	Report numbers of outcome events or summary measures over time	12
Main results	16	(*a*) Give unadjusted estimates and, if applicable, confounder-adjusted estimates and their precision (eg, 95% confidence interval). Make clear which confounders were adjusted for and why they were included	12
		(*b*) Report category boundaries when continuous variables were categorized	NA
		(*c*) If relevant, consider translating estimates of relative risk into absolute risk for a meaningful time period	NA
Other analyses	17	Report other analyses done—eg analyses of subgroups and interactions, and sensitivity analyses	12-14
**Discussion**			
Key results	18	Summarise key results with reference to study objectives	15
**Limitations**			
Interpretation	20	Give a cautious overall interpretation of results considering objectives, limitations, multiplicity of analyses, results from similar studies, and other relevant evidence	15-18
Generalisability	21	Discuss the generalisability (external validity) of the study results	18
**Other information**			
Funding	22	Give the source of funding and the role of the funders for the present study and, if applicable, for the original study on which the present article is based	20

*Give information separately for cases and controls in case-control studies and, if applicable, for exposed and unexposed groups in cohort and cross-sectional studies.

Note: An Explanation and Elaboration article discusses each checklist item and gives methodological background and published examples of transparent reporting. The STROBE checklist is best used in conjunction with this article (freely available on the Web sites of PLoS Medicine at http://www.plosmedicine.org/, Annals of Internal Medicine at http://www.annals.org/, and Epidemiology at http://www.epidem.com/). Information on the STROBE Initiative is available at www.strobe-statement.org.

For peer review only

BMJ Open

Short-term outcomes after emergency surgery for complicated peptic ulcer disease from the UK National Emergency Laparotomy Audit: a cohort study.

Journal:	*BMJ Open*
Manuscript ID	bmjopen-2018-023721.R1
Article Type:	Research
Date Submitted by the Author:	02-Jul-2018
Complete List of Authors:	Byrne, Benjamin; University of Bristol, Centre for Surgical Research, Population Health Sciences; University Hospitals Bristol NHS Foundation Trust, Department of Surgery<br>Bassett, Michael; Royal College of Anaesthetists, National Emergency Laparotomy Audit; Manchester University NHS Foundation Trust, Department of Anaesthesia<br>rogers, chris; University of Bristol, Clinical Trials and Evaluation Unit<br>Anderson, Iain; Royal College of Anaesthetists, National Emergency Laparotomy Audit; Salford Royal NHS Foundation Trust, Department of Surgery<br>Beckingham, Ian; Nottingham University Hospitals NHS Trust, Department of Surgery; Royal College of Surgeons of England, Association of Upper Gastrointestinal Surgeons<br>Blazeby, Jane; University of Bristol, Centre for Surgical Research, Population Health Sciences; University Hospitals Bristol NHS Foundation Trust, Department of Surgery
**Primary Subject Heading**:	Surgery
Secondary Subject Heading:	Epidemiology, Gastroenterology and hepatology, Health services research
Keywords:	EPIDEMIOLOGY, Gastroduodenal disease < GASTROENTEROLOGY, Clinical audit < HEALTH SERVICES ADMINISTRATION & MANAGEMENT, Quality in health care < HEALTH SERVICES ADMINISTRATION & MANAGEMENT, SURGERY

SCHOLARONE™
Manuscripts

Short-term outcomes after emergency surgery for complicated peptic ulcer disease from the UK National Emergency Laparotomy Audit: a cohort study.

B E Byrne[1], M Bassett[2,3], C A Rogers[4], I D Anderson[2,5], I Beckingham[6,7], J M Blazeby[1,8] for the National Emergency Laparotomy Project Team and on behalf of the Association of Upper Gastrointestinal Surgeons

[1]Centre for Surgical Research, Population Health Sciences, Bristol Medical School, University of Bristol, Bristol, UK.

[2]National Emergency Laparotomy Audit, The Royal College of Anaesthetists, London, UK.

[3]Department of Anaesthesia, Manchester University NHS Foundation Trust, Manchester, UK.

[4]Clinical Trials and Evaluation Unit, Bristol Medical School, University of Bristol, Bristol, UK.

[5]Department of Surgery, Salford Royal NHS Foundation Trust, Salford, UK.

[6]Department of Surgery, Nottingham University Hospitals NHS Trust, Nottingham, UK.

[7]Association of Upper Gastrointestinal Surgeons, The Royal College of Surgeons of England, London, UK.

[8]Department of Surgery, University Hospitals Bristol NHS Foundation Trust, Bristol, UK.

Correspondence: B E Byrne

Address: Centre for Surgical Research, Population Health Sciences, Bristol Medical School, University of Bristol, Canynge Hall, 39 Whatley Road, Clifton, Bristol BS8 2PS, UK.

Email: benbyrne@doctors.org.uk.

Telephone: 0117 928 7279

Word count: 3243

For peer review only

For peer review only - http://bmjopen.bmj.com/site/about/guidelines.xhtml

ABSTRACT

Objectives: This study used national audit data to describe current management and outcomes of patients undergoing surgery for complications of peptic ulcer disease, including perforation and bleeding. It was also planned to explore factors associated with fatal outcome after surgery for perforated ulcers. These analyses were designed to provide a thorough understanding of current practice and identify potentially modifiable factors associated with outcome as targets for future quality improvement.

Design: National cohort study using National Emergency Laparotomy Audit (NELA) data.

Setting: English and Welsh hospitals within the National Health Service.

Participants: Adult patients admitted as an emergency with perforated or bleeding peptic ulcer disease between December 2013 and November 2015.

Interventions: Laparotomy for bleeding or perforated peptic ulcer.

Primary and secondary outcome measures: The primary outcome was 60-day in-hospital mortality. Secondary outcomes included length of postoperative stay, readmission and reoperation rate.

Results: 2444 and 382 procedures were performed for perforated and bleeding ulcers, respectively. In-hospital 60-day mortality rates were 287/2444 (11.7%, 95% confidence intervals (CI) 10.5–13.1%) for perforations, and 68/382 (17.8%, CI 14.1–22.0%) for bleeding. Median (interquartile range) 2-year institutional volume was 12 (7-17) and 2 (1-3) for perforation and bleeding, respectively. In the exploratory analysis, age, American Society of Anesthesiology score and pre-operative systolic blood

For peer review only - http://bmjopen.bmj.com/site/about/guidelines.xhtml

For peer review only

pressure were associated with mortality, with no association with time from admission to operation, surgeon grade or operative approach.

Conclusions: Patients undergoing surgery for complicated PUD face a high 60-day mortality risk. Exploratory analyses suggested fatal outcome was primarily associated with patient rather than provider care factors. Therefore, it may be challenging to reduce mortality rates further. NELA data provides important benchmarking for patient consent and has highlighted low institutional volume and high mortality rates after surgery for bleeding peptic ulcers as a target for future research and improvement.

For peer review only

ARTICLE SUMMARY

Strengths and limitations of this study

- This multicentre study examined usual clinical practice across a large number of hospitals in the National Health Service in England and Wales, representing the largest study of complicated peptic ulcer disease yet reported in the United Kingdom.
- Structured data was collected prospectively, mitigating against bias associated with retrospective study design.
- However, case ascertainment within the entire National Emergency Laparotomy Audit patient cohort, the main dataset from which the current study data was extracted, was 83% and missing data may have introduced unknown biases into the study.

For peer review only

INTRODUCTION

Surgical treatment of peptic ulcer disease (PUD) has changed markedly over recent years. Overall operative intervention has declined, with a substantial fall in elective procedures such as gastric resection, vagotomy and pyloroplasty[1–3]. The role of surgery is now largely restricted to the emergency setting, for management of the complications of PUD[4]. Surgical repair is the treatment of choice for perforated PUD and is a second- or third-line treatment for bleeding ulcers that cannot be managed by endoscopic and/or radiological means. Earlier studies and nationwide audits show postoperative mortality following emergency surgery for perforated or bleeding ulcers to range from 9.1% to 26.5% although data from contemporary UK practice is lacking[2,5–8].

The National Emergency Laparotomy Audit (NELA) is a mandatory audit that captures rich data about the care of patients undergoing a range of emergency bowel operations in England and Wales. NELA was established in 2012 and is run by the Royal College of Anaesthetists, in collaboration with the Clinical Effectiveness Unit at the Royal College of Surgeons of England. The audit aims to improve the quality of care for patients undergoing emergency laparotomy, by collecting information on patients, the processes of care they receive and their short-term outcomes. These data are fed back locally, as well as being analysed nationally, and compared against accepted audit standards. To date, there have been three audit reports, most recently documenting a 30-day post-operative mortality rate of 10.6% (95% confidence intervals (CI) 10.2% – 11.0%) and a median length of stay of 11 days across the range of patients and conditions included[9–11].

The present study aimed to use NELA data to identify patients undergoing surgery for perforated or bleeding PUD, to describe the latest management and short-term outcomes for these patients. The study also explored factors that may be associated with mortality after surgery for perforated PUD.

For peer review only - http://bmjopen.bmj.com/site/about/guidelines.xhtml

A thorough appraisal of current practice is critical for benchmarking performance, and appropriately directing future research and quality improvement.

For peer review only

For peer review only - http://bmjopen.bmj.com/site/about/guidelines.xhtml

METHODS

The NELA database contains information collected at the level of individual operations for patients, covering details of the admission, pre-operative management and risk-stratification, intra-operative details, post-operative risk and patient outcomes. From this database, patients aged 18 years or over undergoing 'Peptic ulcer – suture or repair of perforation' or 'Peptic ulcer – oversew of bleed' as their first, main surgical procedure after admission, between 1st December 2013 and 30th November 2015, were selected for inclusion. Re-operations and patients undergoing 'Gastric surgery – other', which is likely to have included formal surgical resection, were excluded. Data for the first and second years of the study were extracted on 1st February in 2015 and 2016, respectively. Data on age, sex, American Society of Anesthesiologists (ASA) score, pre-operative heart rate (HR), pre-operative systolic blood pressure (SBP), pre-operative predicted mortality (P-POSSUM)[12] and morbidity (POSSUM) were extracted[13]. NELA specifies recording of HR and SBP values closest to the time of booking the patient for theatre. Pre-operative care details, including the use of Computed Tomography (CT), time from admission to operation, time from admission to decision to operate, time from decision to operate to operation, and time from admission to antibiotics were also recorded. Information on the grade of most senior operating surgeon and surgical approach (open or minimal access), as well as intra-operative findings (extent and type of peritoneal contamination, see supplementary table 1), were examined, along with the immediate postoperative level of care (ward, level 2 or high dependency unit (HDU), and level 3 or intensive care unit (ICU)) and hospital procedure volume. The following outcomes were examined: total length of stay in an enhanced care setting (HDU or ITU); total postoperative length of stay; return to theatre; and in-hospital death within 60 days of the primary operative procedure.

Statistical analysis

For peer review only - http://bmjopen.bmj.com/site/about/guidelines.xhtml

Patients were grouped and analysed separately according to presentation with perforation or bleeding secondary to PUD. Continuous data were described using mean and standard deviation or median and interquartile range if skewed, and category data were summarised as number and percentage. Length of stay was summarised using survival methods with deaths prior to discharge treated as censored observations.

For the exploratory analysis of patients with perforated PUD, associations between patient, care and operative factors and mortality were examined using multilevel logistic regression, with hospital fitted as random effect. For this analysis alone, only patients undergoing surgery for perforation within 48 hours of admission to hospital were included. This was designed to exclude patients who developed a perforation during their admission, and patients undergoing surgery after an unsuccessful period of non-operative management. Such patients may represent a different population with a different risk profile. For example, severely comorbid patients with mild clinical signs may preferentially be selected for initial non-operative management. The following variables were selected for exploration by consensus within the working group before analysis: age; sex; ASA; HR; SBP; pre-operative CT; time from admission to operation; grade of senior operating surgeon; operative approach; peritoneal contamination type; peritoneal contamination extent; and post-operative care level. Variables with many categories were grouped for analysis (see supplementary table 1). Fractional polynomials were used to describe the relationship between continuous variables and mortality (supplementary table 2). Data that was clearly incorrect was recoded as missing. The analysis was restricted to cases with complete data for the variables of interest. All variables were included in the model and not selected based on statistical significance. All analysis was conducted using Stata 14.0 (StataCorp, College Station, TX).

Ethics

For peer review only - http://bmjopen.bmj.com/site/about/guidelines.xhtml

NELA has approval from the Health Research Authority's Confidentiality Advisory Group for 'Use of Patient Identifiable Information without Consent' (Section 251 of NHS Act 2006 and Health Service (Control of Patient Information) Regulations 2002). The present analysis was performed under NELA's remit to understand and inform the delivery of care to patients undergoing emergency laparotomy. The data extract included anonymised patient level data. Therefore, further approval by a research ethics committee was not required. Participating Trusts follow local governance arrangements for audit registration. Patient data are uploaded via an encrypted website to a secure server. Access is carefully restricted and data used in accordance with the Caldicott principles[14].

Patient involvement

Patients were not involved in any aspect of the design or conduct of this study.

For peer review only - http://bmjopen.bmj.com/site/about/guidelines.xhtml

RESULTS

Patient characteristics and hospital volume

During the study period, 43 321 emergency laparotomies were identified at 192 hospitals in England and Wales. Data on 2444 (5.5%) perforated peptic ulcers and 382 (0.9%) bleeding ulcers were retrieved from 186 (96.9%) contributing hospitals. Patient characteristics are shown in table 1. Over the two-year period, the median number of cases per hospital was 12 (IQR 7-17) and 2 (IQR 1-3) for perforated and bleeding ulcers, respectively.

Table 1. Pre-operative details of patients undergoing surgery for perforation or bleeding.

		Perforation		Bleed		All PUD	
		n=2444	(%)	n=382	(%)	n=2826	(%)
Age in years (mean (SD))	Mean	57.8	(19.4)	65.0	(16.3)	58.8	(19.2)
Sex	Male	1450	(59.3)	240	(62.8)	1690	(59.8)
ASA	1	569	(23.3)	30	(7.9)	599	(21.2)
	2	738	(30.2)	64	(16.8)	802	(28.4)
	3	611	(25.0)	98	(25.7)	709	(25.1)
	4	461	(18.9)	158	(41.4)	619	(21.9)
	5	65	(2.7)	32	(8.4)	97	(3.4)
Pre-op heart rate	<80	449	(18.6)	47	(12.5)	496	(17.8)
	80-99	928	(38.4)	140	(37.1)	1068	(38.2)
	100-119	704	(29.1)	109	(28.9)	813	(29.1)
	120-139	267	(11.0)	65	(17.2)	332	(11.9)
	≥140	69	(2.9)	16	(4.2)	85	(3.0)
Pre-op systolic blood pressure	<80	63	(2.6)	37	(11.6)	100	(3.6)
	80-99	260	(10.8)	96	(30.1)	356	(12.8)
	100-119	670	(27.8)	118	(37.0)	788	(28.3)
	120-139	831	(34.5)	68	(21.3)	899	(32.3)
	140-159	429	(17.8)	43	(11.4)	472	(16.9)
	≥160	157	(6.5)	15	(4.0)	172	(6.2)
Predicted mortality (P-POSSUM)	<5%	935	(38.3)	49	(12.8)	984	(34.8)
	5-9%	416	(17.0)	37	(9.7)	453	(16.0)
	10-24%	445	(18.2)	74	(19.4)	519	(18.4)
	25-49%	292	(11.9)	83	(21.7)	375	(13.3)
	≥50%	356	(14.6)	139	(36.4)	495	(17.5)
Predicted morbidity (POSSUM)	<25%	54	(2.2)	2	(0.5)	56	(2.0)
	25-49%	385	(15.8)	16	(4.2)	401	(14.2)
	50-74%	747	(30.6)	53	(13.9)	800	(28.3)
	≥75%	1258	(51.5)	311	(81.4)	1569	(55.5)

SD – standard deviation; PUD – peptic ulcer disease; ASA – American Society of Anesthesiology score; P-POSSUM – Portsmouth-POSSUM; POSSUM – Physiological and Operative Severity Score for the enUmeration of Mortality and morbidity.

For peer review only - http://bmjopen.bmj.com/site/about/guidelines.xhtml

Pre-operative care

Pre-operative imaging differed according to diagnosis, with the majority (1792 / 2444, 73.3%) of patients undergoing treatment for perforated ulcers receiving a pre-operative CT scan (table 2), compared with 101 / 382 (26.4%) of patients with a bleeding ulcer.

Table 2. Details of pre-operative care of patients undergoing surgery for perforation or bleeding.

		Perforation		Bleed		All PUD	
		n=2444	(%)	n=382	(%)	n=2826	(%)
Pre-operative CT	Yes	1792	(74.1)	101	(26.8)	1893	(67.7)
	No	626	(25.9)	276	(73.2)	902	(32.3)
Time in hours (median (IQR))	Admission to operation	8.8	(5.3-18.9)	30.4	(9.4-107.8)	9.7	(5.5-23.4)
	Admission to decision to operate	6.0	(3.1-14.6)	29.3	(7.5-119.3)	6.5	(3.3-19.4)
	Decision to operate to operation	2.0	(1.2-3.4)	1.1	(0.5-2.1)	1.9	(1.1-3.2)
	Admission to first antibiotics	4.6	(2.1-10.1)	11.8	(3.8-47.8)	5.0	(2.3-11.9)

IQR – interquartile range; PUD – peptic ulcer disease; CT – computed tomography scan.

Median interval from admission to surgery was 8.8 hours (IQR 5.3 – 18.9) in patients with a perforated ulcer, and 30.4 hours (IQR 9.4 – 107.8) in those with a bleeding ulcer. Median interval from decision to operate to surgery was 2.0 (IQR 1.2 – 3.4) and 1.1 (IQR 0.5 – 2.1) hours in patients with perforations and bleeding ulcers, respectively. Patients admitted with a perforated ulcer received their first dose of antibiotics at a median of 4.6 (IQR 2.1 – 10.1; data recorded for 2085 / 2444 (85.3%) of patients) hours after admission, and those undergoing surgery for bleeding ulcers received antibiotics at a median of 11.8 hours (IQR 3.8 – 47.8; data for 273 / 382 (71.5%) of patients).

Operative details and postoperative care level

Consultants were recorded as the senior surgeon in the majority of cases and most surgery was performed via the open approach (table 3). However, 489 / 2 444 (20.0%) patients underwent some

For peer review only - http://bmjopen.bmj.com/site/about/guidelines.xhtml

form of laparoscopic surgery for their perforated ulcer, with 320 (13.1%) procedures completed laparoscopically. The nature and extent of peritoneal contamination differed according to the clinical problem. The majority of patients with perforated ulcers had significant contamination affecting multiple quadrants.

Table 3. Operative details and postoperative destination for patients undergoing surgery for perforation or bleeding.

			Perforation		Bleed		All PUD	
			n=2444	(%)	n=382	(%)	n=2826	(%)
Operation	Senior surgeon	Consultant	1763	(72.1)	347	(90.1)	2110	(74.7)
		Specialty trainee	453	(18.5)	27	(7.1)	480	(17.0)
		Other	228	(9.3)	8	(2.1)	236	(8.4)
	Approach	Open	1955	(80.0)	367	(96.1)	2322	(82.2)
		Laparoscopic (including assisted)	320	(13.1)	10	(2.6)	330	(11.7)
		Laparoscopic converted	169	(6.9)	5	(1.3)	174	(6.2)
	Contamination type	None / minimal	425	(17.4)	250	(65.4)	675	(23.9)
		Significant	2019	(82.6)	132	(34.6)	2151	(76.1)
	Contamination extent	None / single quadrant	753	(30.8)	319	(83.5)	1072	(37.9)
		Multiple quadrants	1691	(69.2)	63	(16.5)	1754	(62.1)
Postoperative care level		Ward (level 1)	1015	(41.5)	68	(17.8)	1083	(38.4)
		HDU (level 2)	652	(26.7)	99	(25.9)	751	(26.6)
		ITU (level 3)	774	(31.7)	212	(55.5)	986	(35.0)

PUD – peptic ulcer disease; HDU – high dependency unit; ITU – intensive therapy unit.

For peer review only

Most patients who underwent surgery to repair a perforated ulcer (1426 / 2444, 58.3%) were transferred to a HDU or ITU environment, with the remainder being transferred to a ward. A much higher proportion of patients operated on for bleeding went to HDU or ITU after surgery (311 / 382, 81.4%).

Outcomes

Among patients transferred to HDU or ITU, the median postoperative stay in an enhanced care environment was 4 days for both groups (table 4). The median total postoperative stay was 8.4 days for patients treated for a perforated ulcer, compared with a longer median stay of 15.0 days for

For peer review only - http://bmjopen.bmj.com/site/about/guidelines.xhtml

bleeding ulcers. The rate of return to theatre was lower among patients operated on for perforation (136 / 2 444, 5.6%) than after surgery for bleeding (36 / 382, 9.4%). In each group, three patients died in theatre. The overall, 60-day in-hospital mortality was 287 / 2 444 (11.7%, 95% CI 10.5% – 13.1%) after surgery for a perforated ulcer, and 68 / 382 (17.8%, 95% CI 14.1% – 22.0%) after oversew of a bleeding ulcer.

Table 4. Outcomes of patients undergoing surgery for perforation or bleeding.

		Perforation n=2 444	(%)	Bleed n=382	(%)	All PUD n=2826	(%)
Length of stay (days)							
HDU/ITU	Median (IQR)	4.0	(2.0-7.0)	4.0	(2.0-8.0)	4.0	(2.0-7.0)
Total	Median (IQR)	8.4	(5.2-18.4)	15.0	(7.5-29.0)	9.2	(5.4-20.0)
Return to theatre		136	(5.6)	36	(9.4)	172	(6.1)
Mortality in-hospital within 60 days		287	(11.7)	68	(17.8)	355	(12.6)
	(Died in theatre)	3	(0.1)	3	(0.8)	6	(0.2)

IQR – interquartile range; PUD – peptic ulcer disease; HDU – high dependency unit; ITU – intensive therapy unit.

Exploratory analysis

Of 2 327 patients where time from admission to operation was recorded, 2 231 (96.1%) underwent surgery for perforation in the first 48 hours after admission. Complete data for regression analysis were available for 2 162 (96.7%) of these patients. Variables identified as significantly associated with in-hospital 60-day mortality (after accounting for other variables) were age, ASA, pre-operative SBP and post-operative care level (table 5). For each increasing year of age, the risk of death rose by 5.0% (95% CI 3.5% – 6.5%), meaning that an increase of 10 years of age was associated with increased risk of death of 63.3% (95% CI 41.6% – 88.4%). An ASA score of 4 or 5 was associated with a markedly elevated risk of fatal outcome compared to an ASA of 1. There was also an increased risk of death for patients going to HDU or ITU compared to those transferred directly to a ward. The association between pre-operative SBP and postoperative mortality was non-linear (illustrated in fig 1). Extremes of low or high SBP were associated with increased risk of death. There was no

For peer review only - http://bmjopen.bmj.com/site/about/guidelines.xhtml

For peer review only

statistically significant association observed between patient sex, pre-operative HR, use of pre-operative CT, time from admission to operation, operating surgeon, operative approach, intra-operative contamination type or extent and subsequent in-hospital mortality.

Table 5. Multilevel logistic regression results, examining factors associated with 60-day in-hospital mortality after surgery for perforation; analysis restricted to patients undergoing surgery within 48 hours of admission.

		Odds ratio	95% confidence intervals		*p*-value
			Lower	Upper	
Age (per year)		1.05	1.04	1.07	<0.001
Sex	Male	1.00			
	Female	1.00	0.70	1.42	0.999
ASA	1	1.00			<0.001
	2	0.77	0.26	2.26	
	3	2.10	0.77	5.76	
	4 & 5	7.19	2.62	19.73	
Pre-op heart rate (per 10 bpm)		1.03	0.95	1.11	0.529
Pre-op systolic blood pressure*					<0.001
Pre-op CT	No	1.00			
	Yes	1.41	0.90	2.22	0.133
Time from admission to operation (per hour)		1.01	0.99	1.02	0.392
Operating surgeon	Consultant	1.00			
	Non-consultant	0.90	0.57	1.40	0.633
Operative approach	Open	1.00			
	Laparoscopic (inc. assisted)	0.78	0.40	1.50	0.459
Contamination type	None / minimal	1.00			
	Significant	0.88	0.49	1.58	0.660
Contamination extent	None / single quadrant	1.00			
	Multiple quadrants	1.14	0.70	1.84	0.605
Postoperative destination	Ward	1.00			
	HDU or ITU	2.22	1.20	4.11	0.011

* - non-linear relationship; ASA – American Society of Anesthesiology score; CT – computed tomography scan; HDU – high dependency unit; ITU – intensive therapy unit.

For peer review only - http://bmjopen.bmj.com/site/about/guidelines.xhtml

DISCUSSION

This is the first national study in the United Kingdom of complicated peptic ulcer disease requiring emergency surgery. Using two years of NELA data, we identified 2444 and 382 patients from 186 English and Welsh hospitals undergoing surgery for perforated or bleeding PUD. The post-operative in-hospital 60-day mortality rates were 11.7% and 17.8%, respectively. In exploratory analysis, mortality after repair of perforated ulcer was primarily associated with patient factors, rather than potentially modifiable aspects of the care provided. This may make it difficult to reduce mortality rates further. Average institutional surgical volume for bleeding ulcers was very low, and these patients had the highest mortality risk, highlighting the challenge and urgent need for further work to understand and improve outcomes in this group.

While mortality rates among the included patients were high, the reported results compare favourably with data from other research. Recent European studies have reported 90-day mortality rates from 19.2% - 29.8% after surgery for perforated PUD[5,15]. Among patients undergoing surgery for bleeding peptic ulcers, 30-day mortality rates were higher, ranging from 23.7% to 25.6%[7,16], with previous UK research revealing a 30% (CI 22% – 38%) post-operative mortality rate[17]. A recent large US study using American College of Surgeons National Surgical Quality Improvement Program data demonstrated similar rates to those observed in this study with 12.1% (CI 10.8% - 13.5%) and 18.6% (CI 15.9% - 21.5%) 30-day mortality after surgery for perforation and bleeding PUD respectively.

The exploratory analysis of factors associated with mortality after repair of perforation generated new, unexpected findings. The significant associations between age, ASA and preoperative SBP and post-operative mortality are unsurprising and consistent with previous research and risk prediction models[18–20]. Accurate and reproducible risk prediction to guide individual patient care would be

For peer review only - http://bmjopen.bmj.com/site/about/guidelines.xhtml

useful but has proven difficult. In this manuscript, our exploratory analysis was principally concerned with a broad assessment of current practice and care provision. We were surprised to find a lack of association between time from admission to surgery and mortality, and this disagrees with the published literature. For example, Buck et al reported that after adjusting for prognostic variables, each hour of surgical delay during the first 24 hours of admission was associated with a 2.4% (CI 1.1 – 3.7%) decrease in the probability of survival[21]. However, that study used less nuanced modelling of continuous variables such as age or shock, which were reduced to dichotomous variables. In addition, they did not describe any exclusion criteria based on time from admission to surgery, raising the possibility that their cohort included patients undergoing surgery after failed conservative management, and those developing a perforation during their hospital admission. The present lack of association between time from admission to operation may have important clinical implications, as it suggests that focusing efforts on reducing the interval from admission to operation may not be the best way to reduce mortality rates. However, pre-operative blood pressure was associated with subsequent mortality, and this is a potentially modifiable variable. Future research and quality improvement should evaluate the role of preoperative optimisation, at the cost of a short delay in transfer to the operating theatre, as a possible strategy to improve outcomes for patients who have already experienced a perforation.

This study found that 13.1% of patients underwent surgical repair of their perforated ulcer via a laparoscopic approach. A further 6.9% were converted from laparoscopic to open. Although the reasons for conversion were not recorded, it is possible that some patients underwent an initial diagnostic laparoscopy before proceeding directly to open repair once the diagnosis was made. A smaller Danish study of 726 patients undergoing surgery for perforated PUD reported a laparoscopy rate of 32.8%, with 24.5% converted from laparoscopic to open[22]. The lower rate in the present study may represent under-reporting, as, anecdotally, some clinicians may not have considered a laparoscopic suture repair eligible for a laparotomy audit. Future comparison with Hospital Episode

For peer review only - http://bmjopen.bmj.com/site/about/guidelines.xhtml
For peer review only

Statistics administrative data, available for all NHS activity, could test this hypothesis. Alternatively, the lower rate may be a true reflection of practice, and lack of skills or confidence in performing laparoscopic repair. The lack of association between operative approach and outcome may suggest that patients are being treated laparoscopically on the availability of appropriately skilled surgeons, rather than through careful case selection or 'picking winners'. Further and more detailed analysis is warranted.

This study has not defined clear ways to improve the survival of the patients included. It has, however, identified aspects of the care provided that were not associated with mortality, suggesting that these should not be the primary focus for immediate quality improvement. The results provide no evidence that more rapid transfer to the operating theatre, greater consultant presence or adoption of minimal access techniques would improve survival rates. However, preoperative SBP may be an appropriate target for future research and quality improvement. Selection of patients for postoperative care in the HDU or ITU environment, and other variables not analysed due to missing data, such as time from admission to antibiotics, should also be investigated further.

The results highlight the low institutional volume for the included procedures, particularly for bleeding PUD requiring surgery. Endoscopy is the first line investigation and treatment for upper gastrointestinal bleeding, and previous large studies have demonstrated the high success rates of endoscopic therapy for bleeding ulcers, with surgery required in 1.9-5.4% of cases[5,16,17]. National guidance in the UK suggests interventional radiology and embolisation should be offered as second-line treatment[23], but few hospitals have 24/7 access to this service. In 2014, 45% of services in England did not have access to either local or networked interventional radiology out of hours[24]. It is likely that many of the 101 of 382 (26.8%) patients with bleeding ulcers that underwent CT had CT angiograms, though the specific details or preoperative CT are not collected in NELA. However, this information would be useful in future updates to the NELA data template. When requiring surgery,

For peer review only - http://bmjopen.bmj.com/site/about/guidelines.xhtml

patients with bleeding ulcers are high risk, as reflected in their ASA scores, with associated high levels of senior involvement in theatre. Low procedure volumes make it difficult to develop expertise managing these patients, which in turn may make it difficult to improve outcomes. In several surgical specialties, higher procedure volume has been associated with improved outcomes[25–27]. However, it is not clear whether such a volume-outcome relationship exists for emergency surgery for bleeding peptic ulcers and it may be difficult to centralise secondary treatment required for an unstable patient. Further research, using quantitative databases and case-studies in different centres, may determine future strategies to improve care for these patients.

This study has several strengths, as a nationwide prospective audit. However, there are limitations. Whilst participation is mandatory, case ascertainment was estimated at 83% in the first two years of the audit[9,10]. No eligible procedures were identified in 6 (3.1%) of hospitals participating in NELA. Patients with complicated PUD that were successfully managed without surgery are not included in NELA. Research in other areas has found that voluntary clinical databases typically demonstrate a lower mortality rate than population-based administrative data[28,29]. This may represent selection bias and it is possible that mortality rates across the country are higher than observed, further highlighting the need for more work in this area. In addition, deaths after discharge, or during the index admission but more than 60 days after the operative procedure, were not included. Another limitation is possible variation in coding of information, which depends upon how different observers interpret the terms. For example, coding of contamination in bleeding ulcers may have reflected existing contamination, or it may have reflected contamination due to the enterotomy required to visualise and treat the bleeding ulcer. It is not possible to retrospectively check the accuracy of such data, which must be taken at face value. While data completeness was satisfactory for the analysis presented, it is not possible to determine whether missing data introduced systematic bias. The extent of missing data precluded exploratory analysis of further variables of interest, such as time from admission to antibiotics, and time from admission to decision to operate.

For peer review only - http://bmjopen.bmj.com/site/about/guidelines.xhtml

While the results may be cautiously generalised to similar populations and healthcare systems, differences in care organisation may limit broad applicability.

In summary, this national study has demonstrated mortality rates within the NHS in England and Wales that compare favourably with previously published international results. The overall rate of mortality, however, remains high. Exploratory analysis suggested fatal outcome after surgery for perforation was primarily associated with patient factors rather than the care provided, and this may make further improvement difficult. As NELA accrues more data over the remaining years of the project, it may be feasible to explore the association between other, modifiable care factors, such as time to antibiotics, and clinical outcomes and this could aid further research. Surgical management of bleeding PUD represents an area of practice with very low volume and high postoperative mortality that mandates further investigation. Centralisation may be considered, though this could be difficult due to the acuity of patients requiring surgery in this setting. Research using future audit data may guide quality improvement efforts, to benefit patients requiring surgery for complications of PUD.

For peer review only

For peer review only - http://bmjopen.bmj.com/site/about/guidelines.xhtml

FIGURE LEGEND

Fig 1. Illustration of non-linear relationship between pre-operative systolic blood pressure and 60-day in-hospital mortality for patients undergoing surgery for perforation only.

For peer review only

For peer review only - http://bmjopen.bmj.com/site/about/guidelines.xhtml

Acknowledgements

The authors would like to thank the members of the NELA Project Team during the data collection period of this study: Mr Martin Cripps, Professor David Cromwell, Dr Emma Davies, Ms Sharon Drake, Dr Mike Galsworthy, Professor Mike Grocott, Dr Angela Kuryba, Mr Jose Lourtie, Dr Ramani Moonesinghe, Dr Dave Murray, Dr Matt Oliver, Mr Dimitri Papadimitriou, Dr Carol Peden, Dr Kate Walker.

Funding

B E Byrne is an academic clinical fellow in surgery supported by the National Institute for Health Research (NIHR). J M Blazeby is a NIHR Senior Investigator. This work was undertaken with the support of the MRC ConDuCT-II (Collaboration and innovation for Difficult and Complex randomised controlled Trials In Invasive procedures) Hub for Trials Methodology Research (MR/K025643/1). The study was also supported by the NIHR Biomedical Research Centre at the University Hospitals Bristol NHS Foundation Trust and the University of Bristol. The views expressed are those of the authors and not necessarily those of the UK National Health Service, National Institute for Health Research, or the Department of Health. The National Emergency Laparotomy Audit is commissioned by the Healthcare Quality Improvement Partnership as part of the National Clinical Audit Programme on behalf of NHS England and the Welsh Government. The funders had no role in study design, data collection and analysis, decision to publish, or preparation of the manuscript.

Competing interests

The authors have declared that no competing interests exist.

For peer review only

For peer review only - http://bmjopen.bmj.com/site/about/guidelines.xhtml

Contributions

IDA, IB and JMB conceived the study. MB and IDA acquired the data. BEB, MB and CAR analysed the data. All authors interpreted the data. BEB drafted the manuscript. MB, CAR, IDA, IB and JMB critically revised the manuscript for important intellectual content. All authors approved the final version for publication.

Data sharing

No additional data available.

Previous presentation

Parts of this work have been presented at the Association of Surgeons of Great Britain and Ireland International Surgical Congress, Glasgow, May 2017, and at the Association of Upper Gastro-Intestinal Surgeons 20th Annual Scientific Meeting, Cork, September 2017.

For peer review only

For peer review only - http://bmjopen.bmj.com/site/about/guidelines.xhtml

REFERENCES

1 Bashinskaya B, Nahed B V, Redjal N, *et al.* Trends in peptic ulcer disease and the identification of Helicobacter Pylori as a causative organism: population-based estimates from the US Nationwide Inpatient Sample. *J Glob Infect Dis* 2011;**3**:366–70.

2 Wang YR, Richter JE, Dempsey DT. Trends and outcomes of hospitalizations for peptic ulcer disease in the United States, 1993 to 2006. *Ann Surg* 2010;**251**:51–8. doi:10.1097/SLA.0b013e3181b975b8

3 Bardhan KD, Williamson M, Royston C, *et al.* Admission rates for peptic ulcer in the Trent Region, UK, 1972-2000. Changing pattern, a changing disease? *Dig Liver Dis* 2004;**36**:577–88. doi:10.1016/j.dld.2004.04.007

4 Paimela H, Oksala NKJ, Kivilaakso E. Surgery for peptic ulcer today: a study on the incidence, methods and mortality in surgery for peptic ulcer in Finland between 1987 and 1999. *Dig Surg* 2004;**21**:185–91. doi:10.1159/000079654

5 Lolle I, Møller MH, Rosenstock SJ. Association between ulcer site and outcome in complicated peptic ulcer disease: a Danish nationwide cohort study. *Scand J Gastroenterol* 2016;**51**:1165–71. doi:10.1080/00365521.2016.1190398

6 Lau JY, Sung J, Hill C, *et al.* Systematic review of the epidemiology of complicated peptic ulcer disease: incidence, recurrence, risk factors and mortality. *Digestion* 2011;**84**:102–13. doi:10.1159/000323958

7 Kyaw M, Tse Y, Ang D, *et al.* Embolization versus surgery for peptic ulcer bleeding after failed endoscopic hemostasis: a meta-analysis. *Endosc Int Open* 2014;**2**:E6–14. doi:10.1055/s-0034-1365235

8 Schroder VT, Pappas TN, Vaslef SN, *et al.* Vagotomy/Drainage is superior to local oversew in patients who require emergency surgery for bleeding peptic ulcers. *Ann Surg* 2014;**259**:1111–8. doi:10.1097/SLA.0000000000000386

For peer review only - http://bmjopen.bmj.com/site/about/guidelines.xhtml

For peer review only

9 NELA Project Team. The first patient report of the national emergency laparotomy audit. 2015.

10 NELA Project Team. The second patient report of the National Emergency Laparotomy Audit. 2016. http://www.nela.org.uk/reports (accessed 18 May 2017).

11 NELA Project Team. Third patient report of the National Emergency Laparotomy Audit. 2017.

12 Prytherch DR, Whiteley MS, Higgins B, *et al.* POSSUM and Portsmouth POSSUM for predicting mortality. *Br J Surg* 1998;**85**:1217–20. doi:10.1046/j.1365-2168.1998.00840.x

13 Copeland GP, Jones D, Walters M. POSSUM: a scoring system for surgical audit. *Br J Surg* 1991;**78**:355–60. doi:10.1002/bjs.1800780327

14 NELA Project Team. Information governance procedures for NELA. http://nela.org.uk/download.php/?fn=Information Governance Procedures for NELA.pdf&mime=application/pdf&pureFn=Information Governance Procedures for NELA.pdf (accessed 18 May 2017).

15 Thorsen K, Søreide JA, Søreide K. Long-term mortality in patients operated for perforated peptic ulcer: factors limiting longevity are dominated by older age, comorbidity burden and severe postoperative complications. *World J Surg* 2017;**41**:410–8. doi:10.1007/s00268-016-3747-z

16 Nykänen T, Peltola E, Kylänpää L, *et al.* Bleeding gastric and duodenal ulcers: case-control study comparing angioembolization and surgery. *Scand J Gastroenterol* 2017;**52**:523–30. doi:10.1080/00365521.2017.1288756

17 Hearnshaw SA, Logan RFA, Lowe D, *et al.* Acute upper gastrointestinal bleeding in the UK: patient characteristics, diagnoses and outcomes in the 2007 UK audit. *Gut* 2011;**60**:1327–35. doi:10.1136/gut.2010.228437

18 Møller MH, Adamsen S, Thomsen RW, *et al.* Preoperative prognostic factors for mortality in peptic ulcer perforation: A systematic review. *Scand J Gastroenterol* 2010;**45**:785–805. doi:10.3109/00365521003783320

For peer review only - http://bmjopen.bmj.com/site/about/guidelines.xhtml

19 Søreide K, Thorsen K, Harrison EM, *et al.* Perforated peptic ulcer. *Lancet* 2015;**386**:1288–98. doi:10.1016/S0140-6736(15)00276-7

20 Møller MH, Engebjerg MC, Adamsen S, *et al.* The Peptic Ulcer Perforation (PULP) score: a predictor of mortality following peptic ulcer perforation. A cohort. *Acta Anaesthesiol Scand* 2012;**56**:655–62. doi:10.1111/j.1399-6576.2011.02609.x

21 Buck DL, Vester-Andersen M, Møller MH. Surgical delay is a critical determinant of survival in perforated peptic ulcer. *Br J Surg* 2013;**100**:1045–9. doi:10.1002/bjs.9175

22 Wilhelmsen M, Møller MH, Rosenstock S. Surgical complications after open and laparoscopic surgery for perforated peptic ulcer in a nationwide cohort. *Br J Surg* 2015;**102**:382–7. doi:10.1002/bjs.9753

23 National Institute For Health and Care Excellence. Acute upper gastrointestinal bleeding in over 16s: management. 2012. https://www.nice.org.uk/guidance/cg141/resources/acute-upper-gastrointestinal-bleeding-in-over-16s-management-pdf-35109565796293

24 Royal College of Radiologists and British Society of Interventional Radiologists. Investing in the interventional radiology workforce: the quality and efficiency case. London: 2014.

25 Birkmeyer JD, Stukel T a, Siewers AE, *et al.* Surgeon volume and operative mortality in the United States. *N Engl J Med* 2003;**349**:2117–27. doi:10.1056/NEJMsa035205

26 Markar SR, Karthikesalingam A, Thrumurthy S, *et al.* Volume-outcome relationship in surgery for esophageal malignancy: systematic review and meta-analysis 2000-2011. *J Gastrointest Surg* 2012;**16**:1055–63. doi:10.1007/s11605-011-1731-3

27 Pieper D, Mathes T, Neugebauer E, *et al.* State of evidence on the relationship between high-volume hospitals and outcomes in surgery: a systematic review of systematic reviews. *J Am Coll Surg* 2013;**216**:1015–25. doi:10.1016/j.jamcollsurg.2012.12.049

28 Almoudaris AM, Burns EM, Bottle A, *et al.* A colorectal perspective on voluntary submission of outcome data to clinical registries. *Br J Surg* 2011;**98**:132–9. doi:10.1002/bjs.7301

29 Aylin P, Lees T, Baker S, *et al.* Descriptive study comparing routine hospital administrative

For peer review only - http://bmjopen.bmj.com/site/about/guidelines.xhtml

data with the Vascular Society of Great Britain and Ireland's National Vascular Database. *Eur J Vasc Endovasc Surg* 2007;**33**:461–5. doi:10.1016/j.ejvs.2006.10.033

For peer review only

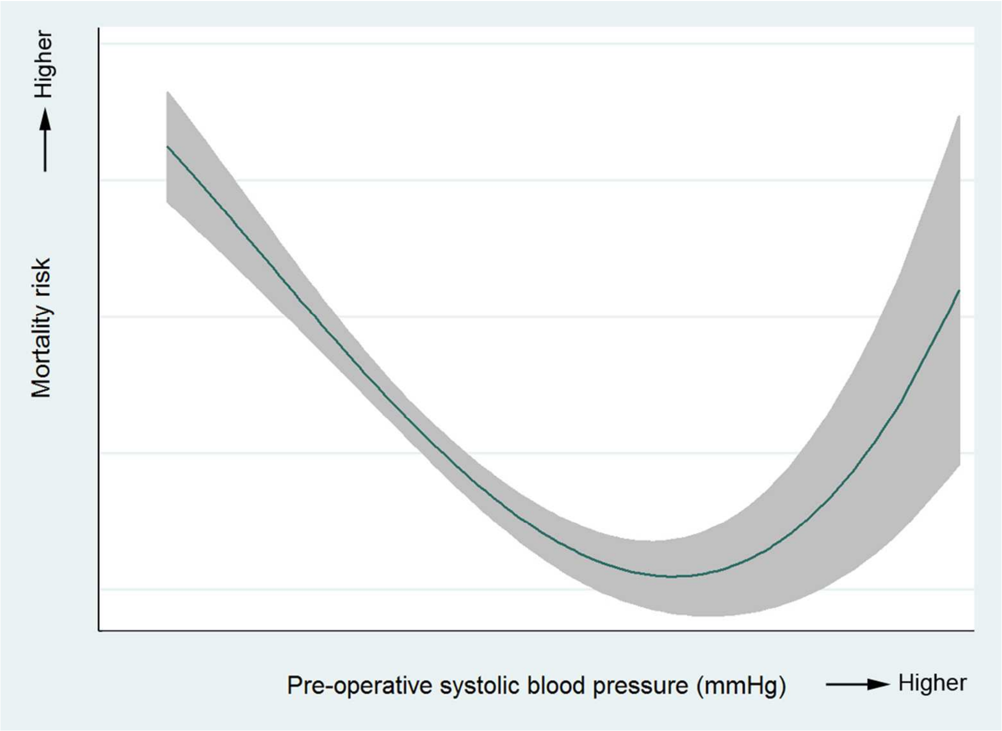

Illustration of non-linear relationship between pre-operative systolic blood pressure and 60-day in-hospital mortality for patients undergoing surgery for perforation only.

101x73mm (300 x 300 DPI)

For peer review only - http://bmjopen.bmj.com/site/about/guidelines.xhtml

SUPPLEMENTARY MATERIAL

Supplementary table 1. Grouping of categorical variables for analysis.

Variable	Group	Original values
ASA	1	1
	2	2
	3	3
	4 & 5	4
		5
Peritoneal contamination type	None / minimal	None / serous
		Gas / minimal
	Significant	Pus
		Bile
		Gastro-duodenal contents
		Small bowel contents
		Faeculent fluid
		Faeces
		Blood
Peritoneal contamination extent	None / single quadrant	None
		Single quadrant
	Multiple quadrants	Multiple quadrants
Preoperative CT	No / missing	No
		Missing
	Yes	Yes
Senior operating surgeon grade	Consultant	Consultant
	Non-consultant	Post CCT fellow
		Specialty trainee
		SAS doctor
		Research/clinical fellow
		Core trainee
		Other
Operative approach	Open	Open
		Laparoscopic converted
	Minimal access	Laparoscopic
		Laparoscopic assisted
Postoperative care level	Ward	Ward
	HDU or ITU	Level 2
		Level 3

ASA – American Society of Anesthesiology score; CT – computed tomography scan; CCT – certificate of completion of training; SAS – specialty and associate specialist doctors.

For peer review only

Supplementary table 2. Relationships between independent variables and mortality.

Variable	Modelling function
Age	Linear
Preoperative HR	Linear
Preoperative SBP	2-term square and cube polynomial
Time admission to decision to operate	Linear

HR – heart rate; SBP – systolic blood pressure.

For peer review only

STROBE 2007 (v4) Statement—Checklist of items that should be included in reports of *cohort studies*

Section/Topic	Item #	Recommendation	Reported on page #
**Title and abstract**	1	(*a*) Indicate the study's design with a commonly used term in the title or the abstract	1
		(*b*) Provide in the abstract an informative and balanced summary of what was done and what was found	2-3
**Introduction**			
Background/rationale	2	Explain the scientific background and rationale for the investigation being reported	5-6
Objectives	3	State specific objectives, including any prespecified hypotheses	5-6
**Methods**			
Study design	4	Present key elements of study design early in the paper	7
Setting	5	Describe the setting, locations, and relevant dates, including periods of recruitment, exposure, follow-up, and data collection	7
Participants	6	(*a*) Give the eligibility criteria, and the sources and methods of selection of participants. Describe methods of follow-up	7
		(*b*) For matched studies, give matching criteria and number of exposed and unexposed	NA
Variables	7	Clearly define all outcomes, exposures, predictors, potential confounders, and effect modifiers. Give diagnostic criteria, if applicable	7
Data sources/ measurement	8*	For each variable of interest, give sources of data and details of methods of assessment (measurement). Describe comparability of assessment methods if there is more than one group	7
Bias	9	Describe any efforts to address potential sources of bias	NA
Study size	10	Explain how the study size was arrived at	NA
Quantitative variables	11	Explain how quantitative variables were handled in the analyses. If applicable, describe which groupings were chosen and why	8
Statistical methods	12	(*a*) Describe all statistical methods, including those used to control for confounding	8
		(*b*) Describe any methods used to examine subgroups and interactions	8
		(*c*) Explain how missing data were addressed	8
		(*d*) If applicable, explain how loss to follow-up was addressed	NA
		(*e*) Describe any sensitivity analyses	NA
**Results**			

For peer review only

Participants	13*	(a) Report numbers of individuals at each stage of study—eg numbers potentially eligible, examined for eligibility, confirmed eligible, included in the study, completing follow-up, and analysed	10
		(b) Give reasons for non-participation at each stage	NA
		(c) Consider use of a flow diagram	NA
Descriptive data	14*	(a) Give characteristics of study participants (eg demographic, clinical, social) and information on exposures and potential confounders	10-11
		(b) Indicate number of participants with missing data for each variable of interest	10-11
		(c) Summarise follow-up time (eg, average and total amount)	13
Outcome data	15*	Report numbers of outcome events or summary measures over time	13
Main results	16	(*a*) Give unadjusted estimates and, if applicable, confounder-adjusted estimates and their precision (eg, 95% confidence interval). Make clear which confounders were adjusted for and why they were included	13
		(*b*) Report category boundaries when continuous variables were categorized	NA
		(*c*) If relevant, consider translating estimates of relative risk into absolute risk for a meaningful time period	NA
Other analyses	17	Report other analyses done—eg analyses of subgroups and interactions, and sensitivity analyses	13-14
**Discussion**			
Key results	18	Summarise key results with reference to study objectives	15
**Limitations**			
Interpretation	20	Give a cautious overall interpretation of results considering objectives, limitations, multiplicity of analyses, results from similar studies, and other relevant evidence	15-19
Generalisability	21	Discuss the generalisability (external validity) of the study results	19
**Other information**			
Funding	22	Give the source of funding and the role of the funders for the present study and, if applicable, for the original study on which the present article is based	21

*Give information separately for cases and controls in case-control studies and, if applicable, for exposed and unexposed groups in cohort and cross-sectional studies.

Note: An Explanation and Elaboration article discusses each checklist item and gives methodological background and published examples of transparent reporting. The STROBE checklist is best used in conjunction with this article (freely available on the Web sites of PLoS Medicine at http://www.plosmedicine.org/, Annals of Internal Medicine at http://www.annals.org/, and Epidemiology at http://www.epidem.com/). Information on the STROBE Initiative is available at www.strobe-statement.org.

For peer review only
